# Supplementary material for: Hfq C-terminal region forms a β-rich amyloid-like motif without perturbing the N-terminal Sm-like structure
Source: Commun Biol. 2023 Oct 21;6:1075. doi: 10.1038/s42003-023-05462-1 (PMC10590398; doi:10.1038/s42003-023-05462-1)
Supplement: Supplementary file 2 — Supplemental Material Berbon et al [file 42003_2023_5462_MOESM2_ESM.pdf]

Supporting information

Hfq C-terminal region forms a β-rich amyloid-like motif without perturbing the N-terminal Sm-like structure

Mélanie Berbon[1], Denis Martinez[1], Estelle Morvan[2], Axelle Grélard[1], Brice Kauffmann[2], Jehan Waeytens[3], Frank Wien[4], Véronique Arluison*[5,6] and Birgit Habenstein*[1]

1 Univ. Bordeaux, CNRS, Bordeaux INP, CBMN, UMR 5248, IECB, Pessac, France
2 Univ. Bordeaux, CNRS, INSERM, IECB, UAR 3033, US001, Pessac, France
3 Laboratoire Léon Brillouin LLB, UMR12 CEA CNRS, CEA Saclay, 91191 Gif-sur-Yvette, France
4 Synchrotron SOLEIL, L'Orme des Merisiers, Saint Aubin BP48, 91192, Gif-sur-Yvette, France
5 Laboratoire Léon Brillouin LLB, UMR12 CEA CNRS, CEA Saclay, 91191 Gif-sur-Yvette, France
6 Université de Paris, UFR SDV, 75013 Paris, France

* Correspondence: V.A. veronique.arluison@univ-paris-diderot.fr; B.H. b.habenstein@cbmn.u-bordeaux.fr

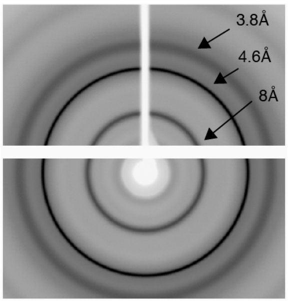

Supplementary Figure 1: X-ray diffraction image of Hfq11 before washing.

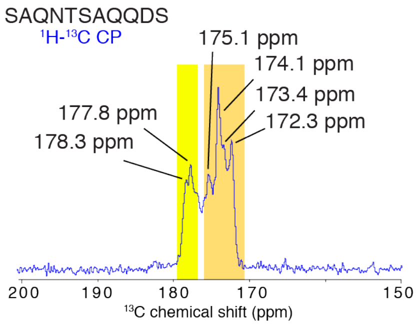

Supplementary Figure 2: ^{1}H-^{13}C cross polarization (CP) NMR spectrum of Hfq11 on a 14.1 T spectrometer at 11 kHz MAS. Peak positions are annotated with their chemical shift values. Highlighted in yellow and orange, respectively, are the spectral regions corresponding to signals of the C=O moieties of side-chain ^{13}C of Asn (N), Asp (D) and Gln (Q) and the backbone C=O moieties of amino acids in β-strand conformation[1].

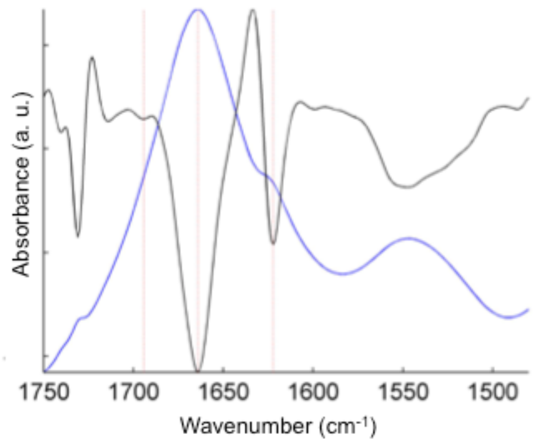

Supplementary Figure 3: ATR-FTIR spectrum of the Hfq11 fibrils before washing.

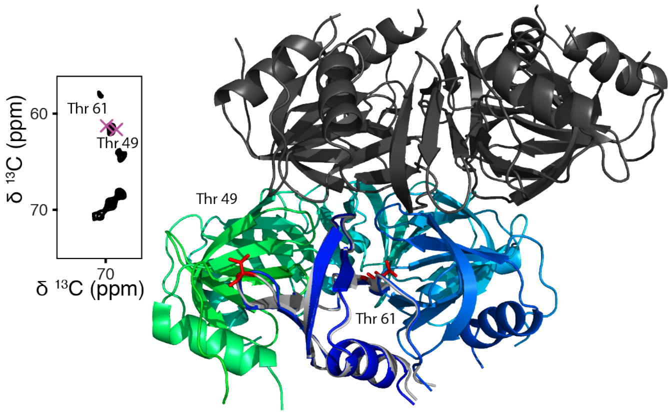

Supplementary Figure 4: ^{13}C-^{13}C PDSD ssNMR spectrum of Hfq fibers, as in Figure 5 in the manuscript. Resonance peaks (violet) of Thr Cα-Cβ correlations, predicted for the Hfq hexamer structure (PDB code: 3QHS, chemical shift predictions from Sparta+[2]), shown on the selected spectral region of Thr signals. Thr residues are highlighted in red on the monomer (grey, PDB code 4RCB) and aligned hexamer (rainbow colour for the aligned hexamer and dark grey for the second hexamer in the asymmetric unit, PDB code 3QHS). Sparta+ chemical shift predictions for PDB code 3QHS and 4RCB are provided in Supplementary Data 1 and 2, respectively.

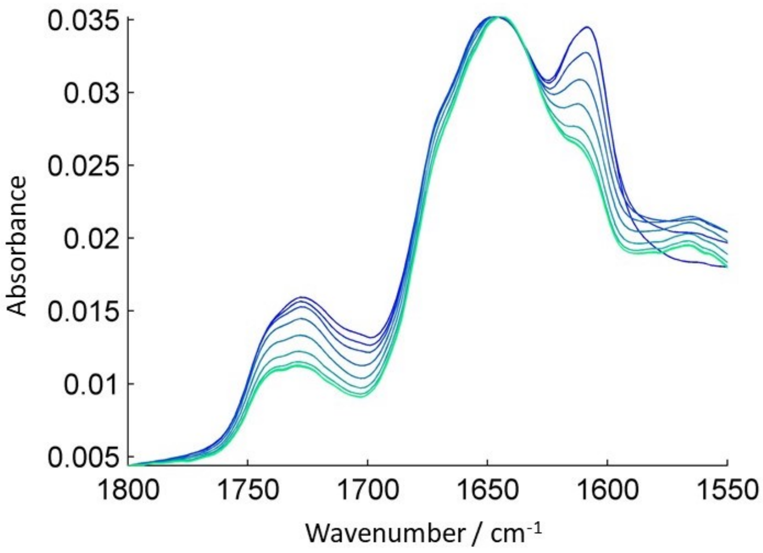

Supplementary Figure 5: FTIR spectra obtained after adding Hfq-CTR fibrils on Dioleoylphosphatidylglycerol (DOPG) lipids. The secondary structure content detected in the eight shown spectra evolves over 12h (from blue to cyan color) with a 90

minutes delay between each spectrum. The decreasing FTIR band at 1610 cm^{-1}, typical for amyloid β-sheets[3], indicates that Hfq CTR fibrils disassemble into a non-amyloid structure.

E. coli	SRPVSH-HSNNA---GGGTSSNYHH-GSSA---QNTSAQQ---DSEETE
S. flexneri	SRPVSH-HSNNA---GGGTSSNYHH-GSSA---QNTSAQQ---DSEETE
S. enterica	SRPVSH-HSNNA---GGGTSSNYHH-GSSA---QNTSAQQ---DSEETE
S. typhimurium	SRPVSH-HSNNA---GGGASNNYHH-GSNA---QGSTAQQ---DSEETE
P. brasilience	SRPVSH-HSNNP----GGS-NNYH--GSNTTA-QQQS-Q----EADDAE
Y. pestis	SRPVSH-HSNTPS----GSTNNYH--GSNPSAPQQP--QQ---DSDDAE
Y. pseudotuberculosis	SRPVSH-HSNTPS----GSTNNYH--GSNPSAPQQP--QQ---DSDDAE
Y. enterocolitica	SRPVSH-HSNNPS----GSTNNYH--GSNPSAPQQP--QQ---DSDDAE
S. proteamaculans	SRPVSH-HSNTPS---GG-TSNYHH-GSNPSAPQQP--QQ---ESDDAE
S. marcescens	SRPVSH-HSNNPS---GG-SSNYHH-GNNPSAQQQP--QQ---ESDDAE
P. septica	SRPVSH-HSNNNT---GGGSNNYHHGGSNASAPSQP--QQ---DSDNAE
E. americana	SRPVSH-HSNNPSS-GGG-S-NYH-QGSTSSAPQQP--QQ---DSDDAE
Y. pseudotuberculosis	SRPVSH-HSNNPS---GS-TNNYH--GSNPSAPQQP--QQ---DSDDAE
C. multitudinisentens	SRPVSH-HSNNPS---G--TSNYHH-GSNPSAPQQP--QQ---ESDDAE
E. carotovora	SRPVSH-HSNNP----GG-SSNNYHHGSNATTAQQQ--SQEA---DDAE
K. Pneumoniae	SRPVSH-HSNNAG---GG-SSNYHH-G--GSAQGSSAPQQ---DSDDAE
A. baumannii	SRPVSH-HSNNAG---GG-SSNYHH-G--GSAQGSSAPQQ---DSDDAE
P. multocida	ARSVSH-H-NNSNNSN---QQNY----------QQE--QQTDSNVEKAE
X. campestris	ARNVRV-GP------GGG----YVQ--------QN-EGGSAG--DDEAE
X. axonopodis	ARNVRV-GP------GGG----YVQ--------QN-EGGSAG--DDEAE
X. fastidiosa	ARNVRV-GP------GGG----YVH------SGSDTL-QIN---DDEVE
N. meningitidis	ARSVNLQHEN---------RPQAAPTSTL---VQV-ETVQQ-----PAE
V. cholerae	ARPVSH-HS--------GDRP----------A--SDRPAEK----SE-E
X. perforans	ARNVRV-GP------GGG----YVQ-SNEGNQAE---------DD-DVEQ
C. crescentus	STIMPA--------------------------Q--PVQLYEPSA--DVDD
B. subtilis	TFAP----------------------------Q----KNVQ----LELE
S. aureus	STYT----VETE-----------A--------Q----------ESTESEE
B. abortus	STIMPSQP-----------V--------QM---------------FEGEEA
C. perfringens	STVSPAKPILFNS----------A--------Q-------------VFDN
P. aeruginosa	SRPVRLP-SGDQPAEPGG---------------------------------NA
P. brassicacearum	VRPIRLP-SATESE--GG----------------------------DAEPGNA

Supplementary Figure 6. Multiple sequence alignment of various bacterial Hfq CTR. The C-terminal regions are variable in length among bacteria and those with a CTR length comparable to that of E. coli Hfq are shown on the top. These CTR usually have a strictly conserved R indicated in pink (R66 in *E. coli*)[4]. CTR aa presumably involved in an amyloid assembly are indicated in blue. Other Hfqs devoid of a long CTR are below. These are usually Gram(+) Hfq, but also in some cases Gram(-), as for C. Crescentus' Hfq. Note that most Hfq CTRs have an acidic tail (in green), in *E.coli* dispensable for amyloid self-assembly[5].

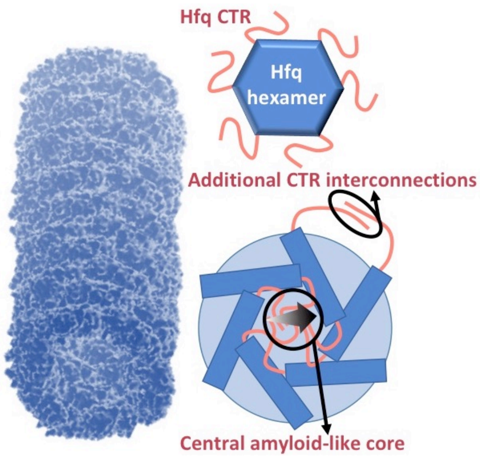

Supplementary Figure 7. Schematic representation of the potential assembly of Hfq hexamers into amyloid-like filaments based on our data and the previously reported electron microscopy data, represented on the left (Reprinted from Journal of Molecular Biology 356 , Arluison et al, "Three-dimensional Structures of Fibrillar Sm Proteins: Hfq and Other Sm-like Proteins », 86-96, Copyright Clearance Center's RightsLink (2006), with permission from Elsevier [6]). For clarity, the CTRs are partially omitted in the scheme of the hexameric assembly. The arrowhead on the lower right panel indicates direction of the β-sheet backbone out of the plane.

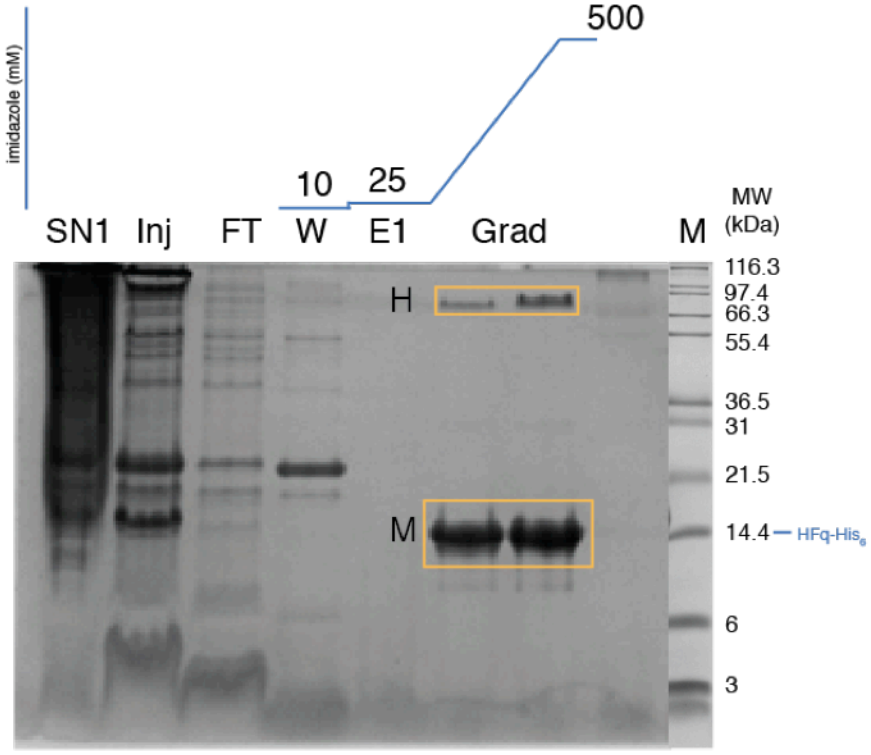

Supplementary Figure 8: Purification gel of ^{13}C-labeled full-length Hfq by HisTrap affinity chromatography. Hfq is eluted in monomeric and hexameric form during imidazole gradient application. Lane 1 : Cell proteins ; Lane 2 : Injection; Lane 3 : FT; Lane 4 : Wash; Lane 5 : E1 25 mM imidazole; Lane 6-7 : linear gradient to 500 mM imidazole; Lanes 11-12 : markers.

Supplementary References

1. Wang, Y. & Jardetzky, O. Probability-based protein secondary structure identification using combined NMR chemical-shift data. *Protein Sci* **11**, 852–61 (2002).
2. Shen, Y. & Bax, A. Protein backbone chemical shifts predicted from searching a database for torsion angle and sequence homology. *J Biomol NMR* **38**, 289–302 (2007).
3. Ruysschaert, J.-M. & Raussens, V. ATR-FTIR Analysis of Amyloid Proteins. in *Peptide Self-Assembly* (eds. Nilsson, B. L.

& Doran, T. M.) vol. 1777 69–81 (Springer New York, 2018).
4. Turbant, F. *et al.* Identification and characterization of the Hfq bacterial amyloid region DNA interactions. *BBA Advances* **1**, 100029 (2021).
5. Malabirade, A. *et al.* Membrane association of the bacterial riboregulator Hfq and functional perspectives. *Sci Rep* **7**, 10724 (2017).
6. Arluison, V. *et al.* Three-dimensional Structures of Fibrillar Sm Proteins: Hfq and Other Sm-like Proteins. *Journal of Molecular Biology* **356**, 86–96 (2006).
