# Supplementary material for: Hfq C-terminal region forms a β-rich amyloid-like motif without perturbing the N-terminal Sm-like structure
Source: Commun Biol. 2023 Oct 21;6:1075. doi: 10.1038/s42003-023-05462-1 (PMC10590398; doi:10.1038/s42003-023-05462-1)
Supplement: Supplementary file 3 — Description of Additional Supplementary Data [file 42003_2023_5462_MOESM3_ESM.docx]

**Description of Additional Supplementary Files**

**File name:** Supplementary Data 1

**Description:** Sparta+ chemical shift predictions for PDB code 3QHS

**File name:** Supplementary Data 2

**Description:** Sparta+ chemical shift predictions for PDB code 4rcb
